# Influence of parental anxiety and beliefs about medicines on feeding and exercise in children living with asthma

**DOI:** 10.1177/13674935231171453

**Published:** 2023-04-25

**Authors:** Rebecca Clarke, Gemma Heath, Prasad Nagakumar, Claire Farrow

**Affiliations:** 1 School of Psychological Science, 1980 University of Bristol, Bristol, UK; 2 School of Psychology, 1722 Aston University, Birmingham, UK; 3 Department of Paediatric Respiratory Medicine and Cystic Fibrosis, 543413 Birmingham Women’s and Children’s Hospital, Birmingham, UK

**Keywords:** Asthma, parents, beliefs about medicines, anxiety, feeding, exercise

## Abstract

This study’s primary objective was to establish differences in beliefs about medicines, levels of asthma-related anxiety and diet and exercise behaviours between parents of children with well controlled and poorly controlled asthma. Secondary objectives were to explore how asthma control might shape relationships between parental cognitions and parenting practices concerning paediatric asthma. Parents of children with asthma aged 10–16 years (*N* = 310) completed standardised questionnaires measuring beliefs about medicines, parental asthma-related anxiety, parenting attitudes towards child activity, parental feeding and asthma control. Parents of children with poorly controlled asthma reported significantly greater asthma medication necessity and concern, asthma-related anxiety, control of child activity, pressure to exercise and unhealthy feeding practices. Moderation analyses indicated that the relationship between parental concern about asthma medicine and parental control of child activity was strongest in children with poorly controlled asthma. Also, the relationship between parental asthma-related anxiety and use of food to regulate child emotion was only significant when asthma was poorly controlled. Parental beliefs about asthma medicines and asthma-related anxiety may indirectly influence asthma outcomes through unhealthy parenting practices around exercise and diet. Eliciting and understanding parents’ perceptions of asthma medications and anxiety may facilitate personalised interventions to improve asthma control.

## Introduction

Asthma is a chronic inflammatory disease of the airways which affects 1.1 million children in the UK and 300 million children globally (Asthma UK, 2017; The National Institute for Health and Care Excellence, 2021). Management aims to control symptoms and prevent asthma attacks and future adverse outcomes (Global Initiative for Asthma: GINA, 2022). Parents play an integral role in monitoring and managing asthma symptoms throughout childhood and adolescence, typically holding responsibility for assessing their child’s breathing, obtaining/administering medicines and supporting their child’s treatment adherence (Koster et al., 2015; Clarke et al., 2021). As their child moves towards adolescence, parents are also responsible for teaching self-management skills and assisting young people to become independent self-carers (Koster et al., 2015; Clarke et al., 2021).

Adherence to asthma medication is crucial to control asthma symptoms (GINA, 2022). Parental beliefs about their child’s treatment can have a significant impact on how they support their child’s illness management. Previous research suggests parents’ beliefs about their child’s medication necessity correlates with adherence behaviour (Sonney et al., 2017). Meanwhile, frequently reported asthma medication concerns (e.g. addiction, immunity and weight gain) (Lycett et al., 2018; Garbutt et al., 2017; De Simoni et al., 2017) have been associated with non-adherence and worse asthma control (Sonney et al., 2017).

Parental concern about harmful consequences of asthma medication or decreasing effectiveness can result in a preference for non-pharmacological approaches to asthma management (Peláez et al., 2017). For example, parents have described reducing their child’s physical activity due to a perception that exercise poses a threat to asthma control (Clarke et al., 2021). As parents report greater exercise limitations in young people with poor asthma control (Clarke et al., 2021; Westergren et al., 2016), it is possible that exercise reduction is perceived to be more important. However, such non-pharmacological approaches can often be inappropriate as dyspnea from exercise intolerance is often incorrectly interpreted by parents as asthma exacerbation (Clarke et al., 2018) and regular exercise engagement can lead to improvements in both exercise capacity and asthma control (Gomes et al., 2015).

Parenting a child who lives with a chronic illness such as asthma, may also have implications for parental wellbeing (Ghaffari et al., 2018; Avcil et al., 2019; Clarke et al., 2021). Higher levels of parental stress have been reported in parents of children with asthma compared with parents of children without asthma (Ghaffari et al., 2018; Avcil et al., 2019). Increased stress and anxiety may result from balancing family and work demands alongside management of a chronic and unstable condition (Avcil et al., 2019). It is possible that parental asthma-related anxiety may interfere with parents’ ability to assist with children’s asthma management (Kish et al., 2018). This may, in part, explain a positive relationship between parental anxiety and poorer asthma outcomes (Avcil et al., 2019).

Theories of parenting have suggested that exposure to chronic stress and anxiety impact practices such as child feeding (El-Behadli et al., 2015). When parents experience greater stress and anxiety, they are more likely to use unhealthy feeding practices, such as exerting more overt control around food or using food as a contingent reward for other behaviours (Hughes et al., 2015; Gouveia et al., 2019). Parents who are busy struggling to cope with daily stressors are thought to be more likely to regulate their child’s emotions by providing food as a time-efficient means of caring and showing support (Hamburg et al., 2014). However, research has yet to explore associations between parental anxiety and feeding in asthma. Given how important diet and weight maintenance are for asthma outcomes (Guillenminault et al., 2017; GINA, 2022), a greater understanding of parental feeding in asthma is necessary. Moreover, better identification of contributors to suboptimal asthma control, such as beliefs about medicines, anxiety and parenting practices, are needed.

## Aim

To establish differences in beliefs about medicines, levels of asthma-related anxiety and parental behaviours between parents of children living with well controlled asthma and those with poorly controlled asthma. Objectives were:

1. To establish whether parents of children with well controlled and poorly controlled asthma report differences in beliefs about medicines, asthma-related anxiety, approaches to physical activity and approaches to child feeding practices.
2. To explore whether asthma control moderates any relationships between:
  a.Parent asthma medication beliefs and control of child activity.
  b.Parent asthma-related anxiety and use of food to regulate a child’s emotions.

## Methods

### Sample and recruitment

Parents of children living with asthma aged between 10 and 16 years were eligible to take part in this study. Parents whose child had a comorbidity that may influence eating or exercise behaviours (e.g. diabetes, irritable bower syndrome and eating disorders) were excluded. Parents of children classified as underweight were also excluded because of likely differences in feeding behaviours related to concerns about underweight. Parent height and weight data were converted to body mass index (BMI) (kg/m^2^). Child height and weight data were converted into standardised BMIz scores from age- and sex-specific data for the UK (Freeman et al., 1990).

Parents were recruited from the UK using an online survey company and reimbursed for their time (Qualtrics). Individuals who had pre-registered interest in taking part in surveys with Qualtrics and had previously reported having a child living with asthma before recruitment began were invited to complete a set of standardised questionnaires between November 2018-February 2019. Recruitment commenced following a favourable ethical opinion by Aston University ethics committee (project #1330). All participants provided informed consent before taking part.

### Measures

Demographic data were collected regarding child age, height and weight, and child ethnicity. The participating parent also reported their own age, height and weight, marital status, education level and household income. Parents also completed the following measures (see Supplementary File 1):

#### Beliefs about medicines

To measure parents’ perceptions of their child’s asthma medication, the Beliefs about Medicines Questionnaire (BMQ: Horne et al., 1999) was used. The BMQ contains two parts: 1) *BMQ-General*, 2) *BMQ-Specific*. The *BMQ-General* consists of two subscales addressing beliefs about medication harm and beliefs about medication overuse. The *BMQ-Specific* scale was adapted to measure parents’ beliefs about necessity of their child’s asthma medication and concerns about potential side effects of asthma medication. Participants responded on a 5-point Likert score to statements such as ‘my child’s life would be impossible without their asthma medication’ and ‘my child having to take asthma medication worries me’. Higher scores indicated a higher perception of particular beliefs or concerns. Cronbach’s alpha scores indicated good internal consistency for general harm, general overuse, specific concerns and specific necessity subscales (α = .81 – .85). The BMQ has been widely used to assess parental beliefs about medicines in children living with asthma (Sonney et al., 2017).

#### Parent asthma-related anxiety

Parental anxiety about their child’s asthma was measured using the Parental Asthma-Related Anxiety Scale (PAAS) (Bruzzese et al., 2011). Currently, the PAAS is the only illness-related anxiety scale to measure specific concerns a parent may have about their child’s asthma. Two subscales were used to measure parental asthma-related anxiety; 1) anxiety related to illness *severity and treatment* (e.g. ‘in the last 2 weeks, how often did you become nervous or worried about your child having an asthma attack and not having his/her asthma medication’), and 2) anxiety related to *disease-related restrictions* (e.g. ‘in the last 2 weeks, how often did you become nervous or worried about your child having an asthma attack when doing physical activity like sports, dancing or exercise’). Participants responded to a 6-point Likert scale to record how nervous or worried they felt about their child’s asthma over the last 2 weeks. A higher score indicated greater parental asthma-related anxiety. Cronbach’s alpha scores were .91 and .90, respectively, indicating a good internal consistency. Previous research has used the PAAS to measure parental asthma-related anxiety (Bruzzese et al., 2016).

#### Parenting practices around child activity

The Parenting Related to Activity Measure (PRAM: Haycraft et al., 2015) was used to explore parenting behaviours of child activity. Three subscales were used to measure: 1) *responsibility and monitoring* (e.g. ‘how often are you responsible for monitoring how much time your child spends engaged in physical activities’, 2) *pressure to exercise* (e.g. ‘my child should always engage in physical activities’, and 3) *control of active behaviours* (e.g. ‘I have to be sure that my child does not engage in too much physical activity’). Participants responded on a 5-point Likert scale with higher scores indicating a greater amount of the particular behaviour or attitude. Cronbach’s alpha scores showed acceptable to good internal consistency (α = .67 - .87).

#### Parental feeding practices

Parental feeding practices were measured using the Comprehensive Feeding Practices Questionnaire (CFPQ); Musher-Eizenman and Holub, 2007). Parents responded using a Likert scale from 1 to 5 for five subscales: 1) *child control of eating*, 2) *use of food for emotion regulation*, 3) *use of food as a reward*, 4) *restriction of food for weight control* and 5) *restriction of food for health*. Questions included those such as ‘I offer sweets (candy, ice cream, cake, pastries) to my child as a reward for good behaviour’ and ‘I restrict the food my child eats that might make him/her fat’. Higher mean scores indicated greater levels of engagement in the particular feeding practice. In this sample, Cronbach’s alpha scores ranged from .69 to .82 demonstrating acceptable to good internal consistency. The CFPQ has previously been validated in an adolescent sample aged from 12 to 17 years (Fleary and Ettienne, 2019).

#### Asthma control

To measure paediatric asthma control, the Asthma Control Questionnaire (ACQ: Juniper et al., 2001) was used. Participants were asked to recall their child’s asthma symptoms over the last week using a 7-point Likert scale (e.g. ‘In general, during the past week how much of the time did you wheeze?’). A higher score indicated poorer asthma control. Following GINA guidelines, a total score of >1.50 was used to categorise poorly controlled asthma in this research (Olaguibel et al., 2012). A total score of <1.50 comprised of the partially controlled and well controlled comparison group, of which <.75 indicated well controlled asthma (Olaguibel et al., 2012). The ACQ is a measure increasingly being used in asthma research with children aged from 6 to 17 years and shows significant correlation with asthma rescue medication use and urgent medical care assistance (Nguyen et al., 2014). Cronbach’s alpha score indicated an excellent internal consistency (α = .93).

### Statistical analysis

Normality tests indicated that the data were not normally distributed. Therefore, non-parametric tests were used for analysis, which where possible with SPSS Statistics V25. A criterion alpha of *p* < 0.05 was set to establish significance. First, Mann–Whitney U and Chi square tests were used to compare participant characteristics between the well-controlled and poorly controlled asthma groups to establish any significant covariates that needed to be controlled for. Following this, Mann–Whitney U tests were used to compare participants’ beliefs about medicines, parental asthma-related anxiety, parenting of child activity and parental feeding practices between both groups.

Finally, moderation analyses were conducted using the PROCESS macro for SPSS (Hayes, 2013) and significant covariates were controlled for. Although the moderation assumption of normality has been violated, this is deemed acceptable for large sample sizes (Hayes, 2013). The current sample size can be considered sufficient, as evidenced by a power analysis. G*Power (Faul et al., 2009) suggests a sample size ≥77 for a small effect size of f2 = 0.15 in a multiple regression with three predictors, using an alpha level of *p* < 0.05 with a statistical power of 0.80.

Four moderation models were tested using Model 1 to assess the moderating effect of asthma control (moderating variable ‘M’) between an antecedent variable ‘X’ and an outcome variable ‘Y’. Moderation analysis was used to explore the relationship between parents’ beliefs about medicines (X) and the parenting of child activity (Y) was moderated by asthma control, such that parental medication concerns predicted less support for activity when asthma was poorly controlled (see Supplementary File 2). Further moderation analysis was used to examine whether the relationship between parental asthma-related anxiety (X) and the *use of food to regulate emotions* (Y) was moderated by asthma control, such that the relationship was stronger when asthma was poorly controlled (see Supplementary File 3). The 95% confidence interval (CI) was used in this analysis to indicate statistical significance.

## Results

### Demographic information

Questionnaires were completed by parents of children living with well controlled (*n* = 181) and poorly controlled asthma (*n* = 129). Data were removed from nine parents who had failed an attention check question or completed the survey in less than 6 min. To ensure accuracy, only weight and height data measured 6 months prior to data collection were used to calculate BMI (*n* = 194). Demographic characteristics of parents are described in Table 1, and characteristics of their children are described in Table 2.

**Table 1.** Socio-demographic characteristics of parent participants.

	Well controlled asthma (n = 181)	Poorly controlled asthma (n = 129)
Parent age (median, IQR)	41 (13.5)	40 (11)
Parent gender (% female)	121 (66.9)	84 (65.1)
Parent BMI (median, IQR)	25.48 (7.29)	27.27 (8.17)
Parent marital status (%)		
Single	28 (15.4)	14 (10.9)
Married	110 (60.8)	80 (62.0)
Co-habiting	30 (16.6)	22 (17.1)
Divorced	10 (5.5)	11 (8.5)
Widowed	0 (0.0)	1 (0.8)
Other	3 (1.7)	1 (0.8)
Parent education status (%)		
To age 16	38 (21.0)	17 (13.2)
AS-Level/A-Level/Other equivalent	54 (29.8)	41 (31.8)
Apprenticeship	13 (7.2)	10 (7.8)
Bachelor’s degree	51 (28.2)	35 (27.1)
Postgraduate degree	20 (11.0)	24 (18.6)
Education level not specified	5 (2.8)	2 (1.6)
Parent employment status (%)		
Unemployed	7 (3.9)	9 (7.0)
Homemaker	35 (19.3)	19 (14.7)
Full time employment	80 (44.2)	83 (64.3)
Part time employment	37 (20.4)	8 (6.2)
Self-employed	12 (6.6)	7 (5.4)
Retired	1 (0.6)	1 (0.8)
Student	1 (0.6)	1 (0.8)
Other	8 (4.4)	1 (0.8)
Parent ethnicity (%)		
White British	161 (89.0)	85.2 (85.3)
Other	20 (11.0)	14.7 (14.7)
Household income (%)		
Less than £10,000	5 (2.8)	5 (3.9)
£10,000–£19,999	34 (18.8)	16 (12.4)
£20,000–£29,999	42 (23.3)	29 (22.5)
£30,000–£39,999	27 (14.9)	25 (19.4)
£40,000–£49,999	29 (16.0)	13 (10.1)
£50,000–£74,999	27 (14.9)	26 (20.2)
£75,000–£99,999	6 (3.3)	11 (8.5)
Over £100,000	5 (2.8)	3 (2.3)
Prefer not to say	6 (3.3)	1 (0.8)

*Note*: BMI: Body Mass Index, IQR: Interquartile Range.

**Table 2.** Socio-demographic characteristics of children.

	Well controlled asthma (*n* = 181)	Poorly controlled asthma (*n* = 129)
Child age (median, IQR)	13 (4)	13 (4)
Child gender (% female)	74 (40.9)	55 (42.6)
ACQ total score (mean, IQ)	0.68 (.42)	2.33 (.67)
Child BMI z-score (median, IQR)	0.88 (2.21)	1.32 (2.09)
Child comorbidities (%)	48 (26.5)	31 (24.0)

*Note*: ACQ: Asthma control Questionnaire, BMI: Body Mass Index, IQR: Interquartile Range.

Parents of children living with poorly controlled asthma reported a significantly higher ACQ total score compared with parents of children living with well controlled asthma: U = 16290, *p* = .01. Parental BMI was also significantly different between parents with well controlled and poorly controlled asthma: U = 9935, *p* = .03. Parents of children living with poorly controlled asthma reported a significantly higher BMI than parents of children living with well controlled asthma. In addition, child BMIz scores significantly differed between groups: U = 3874, *p* = .04. Parents of children living with poorly controlled asthma reported a greater child mean BMIz score than parents of children living with well controlled asthma. Furthermore, Chi square tests indicated that parental employment status was significantly different between participant groups (x^2^(6) = 18.74, *p* = .01). There were no other significant differences in demographics between groups. As a result, parent and child BMI and parental employment status were controlled for during moderation analysis.

### Differences between parental beliefs about medicines, asthma-related anxiety, parenting of child activity and feeding practices between well controlled and poorly controlled asthma

Parents of children living with poorly controlled asthma reported statistically significant greater specific concern and specific necessity beliefs about asthma medication than parents of children living with well controlled asthma (see Table 3). Significantly higher anxiety regarding illness *severity and treatment and disease-related restrictions* were also found in parents of children living with poorly controlled asthma. For parenting practices regarding child activity, significant higher levels of *pressure to exercise* and *control of active behaviours* were reported by parents of children living with poorly controlled asthma. For parental feeding practices; child control, emotion regulation, restriction for weight control, food as a reward and restriction for health were all significantly higher in parents of children living with poorly controlled asthma. No other statistically significant differences were found between other measures for beliefs about medicines or parenting practices (see Table 3).

**Table 3.** Descriptive statistics and Mann–Whitney U analysis comparing the parental beliefs about medicines, asthma-related anxiety, parenting of child activity and parental feeding practices between participants with children with well controlled and poorly asthma.

	Well controlled asthma – Median (IQR)	Poorly controlled asthma – Median (IQR)	Mann–Whitney U tests
Beliefs about medicines			
Necessity of asthma medication	16 (5)	19 (5)	4774**
Concerns about asthma medication	13 (5.5)	17 (5)	5301**
Concerns around general medicine overuse	12 (5)	12 (5)	10929
Concerns about general medicine harm	10 (4)	11 (5)	10380
Parent asthma-related anxiety about:			
Severity and treatment	0.83 (1.17)	1.83 (1.66)	4506**
Disease-related restrictions	1 (1.4)	2 (1.4)	4543**
Parenting practices around child activity			
Responsibility/monitoring	3.5 (1.2)	3.67 (0.91)	10543
Pressure to exercise	3.67 (1.33)	4 (1.34)	8803**
Control of active behaviours	2 (1.34)	2.67 (2)	8131**
Parental feeding practices			
Child control of feeding	2.6 (1)	2.8 (1)	9394*
Use of food for emotion regulation	2 (1.34)	2.33 (0.66)	8513**
Use of food as a reward	2.33 (1.33)	3.33 (2)	7618**
Restriction of food for weight control	3 (1.33)	3.37 (1)	8212**
Restriction of food for health	3.5 (1.5)	3.75 (1)	8685**

*p < .05, **p < .01.

*Note*: IQR: Interquartile Range.

### Asthma control as a moderator of parental beliefs about medicines and parenting around child activity

It was hypothesised that the asthma control group (well controlled/poorly controlled) would moderate the relationship between beliefs about medicines and control of active behaviour. As a significant difference was found between the groups for both BMQ concern and necessity, both subscales were used for analyses. Moderation analysis indicated that the relationship between *concerns about asthma medication* and parental *control of active behaviours* was significantly different for parents of children with well controlled asthma compared to those with poorly controlled asthma; *b* = 1.18, 95% [Cl .15, .2.22], *t* = 2.27. A significant positive relationship between parents’ *concerns about asthma medication* and *control of active behaviours* was found when asthma was well controlled; *b* = .85, 95% [Cl .11, 1.59], *t* = 2.27. However, for the group with poorly controlled asthma, there was an even stronger positive relationship between *concerns about asthma medication* and control of child activity; *b* = 2.04, 95% [Cl 1.32, 2.76], *t* = 5.6.

Further moderation analysis did not find asthma control to moderate the relationship between the *necessity of asthma medication* and parental *control of active behaviours* (*b* = .03, 95% [Cl −.95, 1.01], *t* = .06).

### Asthma control as a moderator between parental asthma-related anxiety and the use of food to regulate emotions

It was hypothesised that asthma control would also moderate the relationship between parental asthma-related anxiety and the use of food to regulate emotions. Moderation analysis indicated that the relationship between parental anxiety about asthma *severity and treatment* and parental *use of food for emotion regulation* was significantly different for parents of children with well controlled asthma compared to those with poorly controlled asthma; *b* = .33, 95% [Cl .09, .58], *t* = 2.73. The relationship was not found to be significant for the group with well controlled asthma; *b* = −.1, 95% [Cl −.28, .08], *t* = −1.06, however, for the group with poorly controlled asthma, greater parental anxiety about asthma *severity and treatment* significantly predicted greater *use of food for emotion regulation*; *b* = .23, 95% [Cl .07, .39], *t* = 2.96.

Moderation analysis also indicated that the relationship between parental anxiety about asthma *disease-related restrictions* and the *use of food for child emotion regulation* was significantly different for parents of children with well controlled asthma compared to those with poorly controlled asthma; *b* = .31, 95% [Cl .06, .56], *t* = 2.47. The relationship was not significant for the group with well controlled asthma; *b* = −.08, 95% [Cl −.25, .09], *t* = −.91, however, for the group with poorly controlled asthma, parental asthma-related anxiety about *disease-related restrictions* significantly predicted higher *use of food for child emotion regulation*; *b* = .23, 95% [Cl .05, .4], *t* = 2.5.

## Discussion

This study aimed to explore relationships between parental beliefs about medicines, asthma-related anxiety and parental practices regarding child activity and feeding, in parents of children living with well controlled and poorly controlled asthma. Parents of children living with poorly controlled asthma reported greater need for asthma medication, greater concern about treatment outcomes and higher parental asthma-related anxiety. There was also greater perceived pressure to exercise and control of child activity as well as greater unhealthy feeding practices reported by parents of children with poorly controlled asthma. The relationship between specific concerns about asthma medicines and parental control of child activity behaviours was stronger in children with poorly controlled asthma. Furthermore, the relationship between parental asthma-related anxiety and use of food to regulate child emotion was only significant when asthma was poorly controlled.

Findings indicate no statistically significant differences between asthma groups’ beliefs about medicines in general. However, in line with previous research, parents of children living with poorly controlled asthma reported significantly greater need for, and concerns about, asthma medication (Koster et al., 2011). The Necessity-Concerns Framework (Horne and Weinman, 1999) suggests that when high necessity and high concern beliefs coincide, people will feel ambivalent towards taking their medication. Such ambivalence may lead to suboptimal adherence behaviours, perhaps explaining why this study found both high medication necessity and high medication concern beliefs by parents of children living with poorly controlled asthma.

Statistically significant greater parental control of child activity and pressure to exercise were reported by parents of children living with poorly controlled asthma. Higher reported control of child activity suggests that perceptions of exercise barriers may increase as asthma control declines (Westergren et al., 2016). This may explain hypervigilant parenting around physical activity in paediatric asthma and a desire to control children’s exercise engagement (Clarke et al., 2021). Similar parenting practices may occur in other paediatric chronic conditions as a means of illness management, as literature suggests that some parents of children living with type 1 diabetes do not promote physical activity to reduce risk of blood glucose fluctuations (Blake et al., 2018).

A greater pressure to exercise was also reported by parents of children living with poorly controlled asthma suggesting that parents also recognised physical activity as essential. It is necessary that families living with paediatric asthma are provided with safe, controlled environments in which to exercise to help reduce concerns, improve risk management and motivate physical activity engagement (Clarke et al., 2021).

Asthma control was also found to moderate a positive relationship between specific concerns about asthma medication and parental control of active behaviours in both groups, yet a stronger relationship was found in families of children with poorly asthma control. Physicians need to be aware of parental beliefs about asthma medicines as they may interfere with exercise recommendation as part of a treatment plan. Previous research has found reduced exercise in children with worse asthma control (Lang, 2014). It is possible that breathlessness from poor aerobic fitness could be interpreted as exercise induced bronchoconstriction and reinforce parental concern about medicines and need for exercise restriction. Supporting families to correctly identify symptoms may help to promote adherence and activity (Clarke et al., 2021).

Identification of parental beliefs may be challenging. It has previously been suggested that parents do not share medication concerns in consultations, and addressing adherence ambivalence can be difficult for clinicians during short consultation times (Unni & Shivanbola, 2016; Abdel-Tawab et al., 2011). Implementation of patient-centred consultation tools may be a time-efficient method to explore a range of factors that can interfere with adherence behaviours. The Medicine-Related Consultation Framework (MRCF) can help healthcare professionals to elicit patient medicine beliefs and create personalised management plans (Abdel-Tawab et al., 2011). Utilising other techniques, such as motivational interviewing (MI), in consultations can help to improve families’ motivation and commitment to treatment adherence (Gesinde and Harry, 2018).

A greater understanding is needed concerning emotional burden experienced by caregivers of children who are living with asthma (Shaw et al., 2020). Findings from this study support previous reports that greater parental anxiety correlates with worse asthma outcomes (Avcil et al., 2019). Although causality in this relationship is unclear, literature suggests that parents of children living with a chronic illness have the highest levels of parenting stress which varies by illness severity (Pinquart, 2017). It is possible that worse asthma outcomes may increase parenting demands, such as greater healthcare utilisation (Ghaffari et al., 2018), and in turn reduce parent’s ability to effectively support young people’s needs (Koster et al., 2015). Alternatively, increased parental anxiety may result from greater distress in young people with poorly controlled asthma (Selby et al., 2018). Family centred support groups could provide benefits to family psychological wellbeing as well as asthma outcomes, although research is needed.

Parents of children living with poorly controlled asthma reported greater child control of diet, use of food to regulate emotions, use of food as a reward and restriction of diet for weight management and health. Higher reports of parental anxiety in this sample may explain use of increased unhealthy feeding practices (El-Behadli et al., 2015). Additionally, reports of increased restriction of food for health and weight management may arise from increased child weight found in this sample alongside knowledge that a healthy diet and weight can improve asthma outcomes (GINA, 2022; Guillenminault et al., 2017). However, this may have negative consequences as previous research suggests restrictive feeding practices and use of food as a reward or to regulate emotions can result in young people struggling to understand how to regulate their appetite and further weight gain (Aparicio et al., 2016; Hamburg et al., 2014).

Poorly controlled asthma was found to moderate the relationship between parental asthma-related anxieties (related to severity and treatment, and disease-related restrictions), and the use of food to regulate emotions. Causality of these relationships cannot be established*.* It is possible that emotional feeding is used as a tool to alleviate parents’ anxieties and perceived higher levels of distress in young people with worse manifestations of asthma (Hamburg et al., 2014). Moreover, parental feeding practices may also be responsive to young people’s demands, as greater binge eating has previously been found in adolescents with increased health dissatisfaction (Olguin et al., 2017).

It is also possible that poor diet quality may increase airway inflammation and worsen asthma control (Guillenminault et al., 2017). Associations between poor diet and worse asthma control emphasise a need to address factors that may influence poor dietary intake. Peer influence on dietary choices should particularly be considered during adolescence as young people move towards independence and responsibility for choosing healthier foods is incrementally transferred to young people themselves. Adolescence is a time when adult health behaviours are established and thus represents a window of opportunity for interventions to promote and sustain adaptive self-management throughout their life (Heath et al., 2016). Addressing parental asthma-related anxiety alongside education around diet may be an important addition to family centred intervention.

### Limitations

To our knowledge, findings of a relationship between parents’ asthma-related anxiety and child feeding, and quantitative reports of a relationship between parental medication beliefs and parenting around child activity in paediatric asthma, are novel. Findings highlight the importance of parenting practices, caregiver’s emotional wellbeing and medication beliefs when considering asthma control. Nevertheless, data is cross-sectional and we cannot therefore determine directionality of relationships. It is likely that correlations found are bi-directional. Future research should use longitudinal designs to establish causality, to deepen our understanding of the correlations and differences established.

Despite parental recall being in line with GINA recommendations to help establish asthma control (GINA, 2022), findings should be interpreted in light of parental reports and use of the CFPQ, a scale developed for a younger population. We recommend that future research includes objective measures, such as physiological assessment or unscheduled healthcare utilisation, to provide more robust measures of asthma diagnosis and control, as well as child BMIz score. It may also be useful to ascertain whether parent participants are the primary caregiver and responsible for providing meals. Furthermore, including young people in future research may support a more comprehensive, robust understanding of how the young patient’s behaviour and beliefs can influence asthma control, particularly as they approach adolescence where responsibility for health behaviours (e.g. food choices, medication adherence) is incrementally transferred and peers take on an increasingly influential role (Koster et al., 2015; Clarke et al., 2021).

This research should also be interpreted in light of its’ homogeneous sample. Future research must recruit families using multiple methods to ensure participants from more diverse backgrounds are included as well as considering wider family psychosocial and socioeconomic factors that can influence parenting. Nevertheless, this study highlights important correlations between parental beliefs about medicines, and parental asthma-related anxiety, and parenting practices, and provides a basis for further investigation.

### Implications for practice

It is crucial to understand practices that parents use in response to their medication beliefs, anxiety and asthma control as they are likely to indirectly influence poorer asthma outcomes through reduced exercise, poor diet quality and increased child weight (Lang, 2014). Strong patient-physician dyads need to be developed alongside use of consultation tools, such as the MRCF, to enhance time-efficient elicitation of beliefs about medicines and parental anxiety (Abdel-Tawab et al., 2011; Tilly-Gratton et al., 2018). Brief interventions and MI during consultations can help to encourage families to adopt behavioural change, such as treatment adherence, in a way that aligns with their own values and goals whilst exploring and resolving patient ambivalence (Gesinde and Harry, 2018). Such family centred approaches will also support young people to develop and maintain healthy behaviours whilst considering beliefs and anxiety as asthma management and self-care is transferred to them.

Providing guidance to improve families’ interpretation of breathlessness during exercise, pulmonary rehabilitation and family illness management programmes are other strategies to help families overcome barriers to exercise and effectively manage anxiety (Westergren et al., 2016; McDonald and Ram, 2017; Van Mechelen et al., 2018). Parents play an important role in promoting and normalising physical activity and healthy eating alongside the correct therapeutic asthma management. Engaging parents of young people with asthma in psycho-educational programmes and behaviour change interventions may lead to positive outcomes for their child’s asthma control.

## Conclusion

Unhealthy parenting practices, such as using food to regulate emotions and control of child activity, can be influenced by parental beliefs about medicines and asthma-related anxiety. Asthma control has been found to moderate relationships between parental asthma-related anxiety and using food to regulate emotions, and concerns around asthma medication and control of child physical activity. These results highlight the importance of eliciting parents’ beliefs about medicines and parental asthma-related anxiety. Parenting practices are modifiable and understanding what may drive them provides a target for intervention to improve paediatric asthma outcomes.

## Supplemental Material

Supplemental Material - Influence of parental anxiety and beliefs about medicines on feeding and exercise in children living with asthma Supplemental Material for Influence of parental anxiety and beliefs about medicines on feeding and exercise in children living with asthma by Rebecca Clarke, Gemma Heath, Prasad Nagakumar and Claire Farrow in Journal of Child Health Care.

Supplemental Material - Influence of parental anxiety and beliefs about medicines on feeding and exercise in children living with asthma Supplemental Material for Influence of parental anxiety and beliefs about medicines on feeding and exercise in children living with asthma by Rebecca Clarke, Gemma Heath, Prasad Nagakumar and Claire Farrow in Journal of Child Health Care

Supplemental Material - Influence of parental anxiety and beliefs about medicines on feeding and exercise in children living with asthma Supplemental Material for Influence of parental anxiety and beliefs about medicines on feeding and exercise in children living with asthma by Rebecca Clarke, Gemma Heath, Prasad Nagakumar and Claire Farrow in Journal of Child Health Care.
