# Supplementary material for: Influence of parental anxiety and beliefs about medicines on feeding and exercise in children living with asthma
Source: J Child Health Care. 2023 Apr 25;28(4):865–79. doi: 10.1177/13674935231171453 (PMC11607838; doi:10.1177/13674935231171453)
Supplement: Supplemental Material - Influence of parental anxiety and beliefs about medicines on feeding and exercise in children living with asthma [file sj-pdf-1-chc-10.1177_13674935231171453.pdf]

Supplementary File 1: Summary of measures used

Measure	Abbreviation	Summary
Asthma Control Questionnaire (Juniper et al., 2001)	ACQ	A measure of asthma control. Higher scores indicate poorer asthma control.
Beliefs about Medicines Questionnaire (Horne et al., 1999)	BMQ	A measure of perceptions about medication in general and specific medications, such as asthma medication. Higher scores indicate stronger concerns and beliefs.
Comprehensive Feeding Practices Questionnaire (Musher-Eizenman et al., 2007)	CFPQ	A measure of feeding practices used by parents. Feeding practices measured included: child control of eating; use of food for emotion regulation; use of food as a reward; restriction of food for weight control; and restriction of food for health. Higher scores indicate a greater amount of the particular child feeding practice.
Parental Asthma-Related Anxiety Scale (Bruzzese et al., 2011)	PAAS	A measure of parent's anxiety about their child's asthma. In particular, anxiety about asthma severity and treatment and anxiety about asthma-related restrictions. A higher score indicates greater asthma-related anxiety.
Parenting Related to Activity Measure (Haycraft et al., 2015)	PRAM	A measure of parenting behaviours and perceptions around child's physical activity. Subscales measured parental responsibility and monitoring of child activity, parental pressure for child to exercise, and parental control of child active behaviours. Higher scores indicate a greater amount of a particular behaviour or attitude.
