# Supplementary material for: Influence of parental anxiety and beliefs about medicines on feeding and exercise in children living with asthma
Source: J Child Health Care. 2023 Apr 25;28(4):865–79. doi: 10.1177/13674935231171453 (PMC11607838; doi:10.1177/13674935231171453)

Supplementary File 3: Conceptual moderation model of the relationship between parental asthma-related anxiety (X) and food for emotion regulation (Y) with asthma control as a moderator (M).

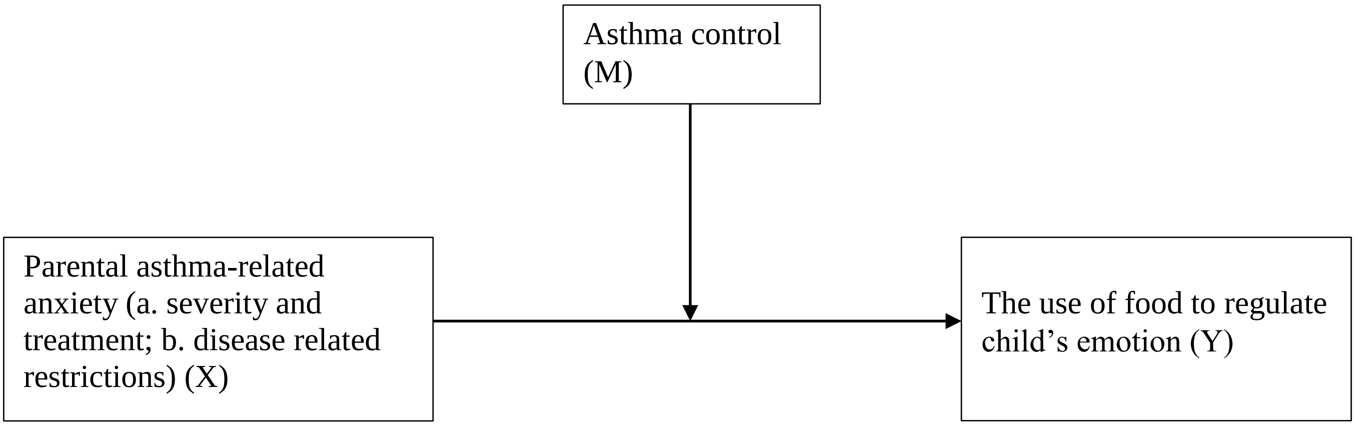

Supplement: Supplemental Material - Influence of parental anxiety and beliefs about medicines on feeding and exercise in children living with asthma [file sj-pdf-3-chc-10.1177_13674935231171453.pdf]
